# Sulfur mustard single-dose exposure triggers senescence in primary human dermal fibroblasts

**DOI:** 10.1007/s00204-022-03346-7

**Published:** 2022-07-29

**Authors:** Gabriele Horn, Catherine Schäfers, Horst Thiermann, Sandra Völkl, Annette Schmidt, Simone Rothmiller

**Affiliations:** 1 Bundeswehr Institute of Pharmacology and Toxicology, Neuherbergstraße 11, 80937 Munich, Germany; 2 Institute of Sport Science, University of the Bundeswehr Munich, Werner-Heisenberg-Weg 39, 85577 Neubiberg, Germany

**Keywords:** Sulfur mustard, Senescence, Human dermal fibroblasts, Wound healing disorder

## Abstract

Chronic wounds, skin blisters, and ulcers are the result of skin exposure to the alkylating agent sulfur mustard (SM). One potential pathomechanism is senescence, which causes permanent growth arrest with a pro-inflammatory environment and may be associated with a chronic wound healing disorder. SM is known to induce chronic senescence in human mesenchymal stem cells which are subsequently unable to fulfill their regenerative function in the wound healing process. As dermal fibroblasts are crucial for cutaneous wound healing by being responsible for granulation tissue formation and synthesis of the extracellular matrix, SM exposure might also impair their function in a similar way. This study, therefore, investigated the SM sensitivity of primary human dermal fibroblasts (HDF) by determining the dose–response curve. Non-lethal concentrations LC_1_ (3 µM) to LC_25_ (65 µM) were used to examine the induction of senescence. HDF were exposed once to 3 µM, 13 µM, 24 µM, 40 µM or 65 μM SM, and were then cultured for 31 days. Changes in morphology as well as at the genetic and protein level were investigated. For the first time, HDF were shown to undergo senescence in a time- and concentration-dependent manner after SM exposure. They developed a characteristic senescence phenotype and expressed various senescence markers. Proinflammatory cytokines and chemokines were significantly altered in SM-exposed HDF as part of a senescence-associated secretory phenotype. The senescent fibroblasts can thus be considered a contributor to the SM-induced chronic wound healing disorder and might serve as a new therapeutic target in the future.

## Introduction

Human dermal fibroblasts (HDF) have an underestimated role in the skin homeostasis (Wlaschek et al. 2021). They represent the most widespread cell type within the dermis and are responsible for the synthesis and organization of extracellular matrix (ECM) and the communication with the adjoining stem cell niche (Wlaschek et al. 2021; Harding et al. 2005; Ho and Dreesen 2021). Crosstalk between fibroblasts and epidermal basal keratinocytes is essential for proliferation of basal stem cells which guarantee formation and differentiation of functional epidermis (Ho and Dreesen 2021; Yang et al. 2019). In case of skin damage, HDF contribute significantly to the process of wound healing starting with the breakdown of fibrin clots and the subsequent formation of collagen structures (Bainbridge 2013). In the final phase of wound healing, HDF are stimulated by growth factors to differentiate into myofibroblasts to induce wound contraction for tissue remodeling (Yang et al. 2019).

Chronic wounds as the result of an impaired skin homeostasis are characterized by defective remodeling of ECM, failure of reepithelialization and prolonged inflammation (Cook et al. 2000). Previous studies demonstrated that HDF derived from chronic wounds show abnormal morphology, a lower proliferation rate, early senescence and an altered secretion of cytokines and growth factors (desJardins-Park et al. 2018; Harding et al. 2005). Due to the functional changes, HDF may no longer be able to undertake their role in the wound healing process. Studies postulate that a critical point for wound healing is reached if 15% of wound skin fibroblasts have turned senescent as they are able to induce senescence in neighboring cell types in a paracrine manner via e.g., their senescence-associated secretory phenotype (SASP), the secretion of a unique repertoire of molecules (Harding et al. 2005; Wilkinson and Hardman 2020). Thus, senescence can be considered as a significant factor in wound healing disorder. Senescence is defined as a stable arrest of the cell cycle that dramatically affects gene and protein expression as well as mitochondrial function or phenotype of cells (Kumari and Jat 2021). It can have a positive effect as part of a physiological response by prevention of excessive fibrosis in cutaneous injury or of proliferation of potential cancer cells but is also involved in the aging process by interfering with tissue repair and regeneration (Kumari and Jat 2021; Wilkinson and Hardman 2020). Various intrinsic and extrinsic factors were identified triggering senescence such as telomere attrition in the replication process, oxidative stress, irradiation or genotoxic substances as DNA-damaging drugs (Gorgoulis et al. 2019). The DNA-damaging substance sulfur mustard (SM), an alkylating chemical warfare agent, is banned by the Chemical Weapons Convention but was recently deployed in a conflict in Syria (John et al. 2019). Upon contact with skin, SM causes edema, inflammation and skin blisters after an asymptomatic period followed by ulcerations as a result of chronic wound healing disorder (Schmidt et al. 2013; Kehe et al. 2009). So far, the signaling pathway of SM is not fully understood. In consequence, there are no specific treatment options, neither antidotes nor a prophylaxis available (Etemad et al. 2019). Recently, Rothmiller et al. (2021) found that SM induces chronic senescence in human mesenchymal stem cells (MSC) which is considered to contribute to the wound healing disorder. As HDF also play a crucial role in wound healing, they might be affected during SM exposure. So far, there have been no data on the effects of SM on HDF. In this study, we investigated the SM sensitivity and the induction of senescence in HDF and provide new insights into the influence of SM on HDF which might contribute to find new therapeutic approaches against SM-induced wound healing disorder.

## Materials and methods

### Cell culture

Human primary dermal fibroblasts (HDF, PCS-201-010) from neonatal foreskin were purchased from ATCC (American Type Culture Collection, Manassas, Virginia, USA). Cells were seeded with corresponding fibroblast basal medium (PCS-201-030), supplemented with fibroblast growth kit—low serum (PCS-201-041). Final concentrations of supplements in complete fibroblast culture medium (FCM) were 7.5 mM l-glutamine 5 ng/mL rh-FGF basic, 5 µg/mL rh-Insulin, 1 µg/mL hydrocortisone, 50 µg/mL ascorbic acid and 2% fetal bovine serum. Cultures were maintained at 37 °C in a humidified atmosphere with 5% CO_2_ in standard culture flasks (25 cm^2^, 75 cm^2^, 175 cm^2^, Greiner AG, Kremsmünster, Austria). FCM was changed every 2–3 days and cells were cultured until 70% confluence. For passaging, cells were washed once with PBS (pH 7.4, 1×, Thermo Fisher Scientific, Waltham, Massachusetts) and trypsinized using 0.05% Trypsin in 1 mM EDTA (Thermo Fisher Scientific, Waltham, Massachusetts) for 5 min at 37 °C. Trypsin was neutralized by adding FCM. Cell suspension was pelleted at 522×*g* for 5 min and HDF were resuspended in FCM followed by determination of the cell number using counting chamber C-Chip Neubauer improved (NanoEnTek Inc, Seoul, Korea). Cells were replated at 5000 cells per cm^2^ in fresh FCM. Experiments in this study were started with HDF up to passage three.

### ***Determination of cell viability after SM and H***_***2***_***O***_***2***_*** exposure***

SM (bis-[2-chloroethyl]sulfide; purity > 99%, confirmed by NMR) was made available by the German Ministry of Defense. Prior to the experiments, SM (8 M) was freshly pre-diluted in ethanol. The 30% (w/w) H_2_O_2_ solution in H_2_O (Sigma-Aldrich, St. Louis, USA) was pre-diluted in ultra-pure water. For experimental approaches, SM or H_2_O_2_ was diluted in FCM. The final ethanol content in SM experiments was 0.25%.

HDF were plated at 20,000 cells per well in two (H_2_O_2_ exposure) or three (SM exposure) 24-well plates (Greiner AG, Kremsmünster, Austria) including medium control and were grown overnight. The next day, cells were exposed to increasing concentrations of H_2_O_2_ (final concentrations 300–2000 μM) or SM (final concentrations 0.03–1000 µM) as well as to solvent control for 24 h. Then, cells were washed once with PBS. Per plate, 5 mL XTT labeling reagent was mixed with 100 μL electron-coupling reagent to prepare XTT staining solution (Sigma-Aldrich, St. Louis, USA). 400 μL FCM and 200 µL XTT staining solution were added to the cells. After incubation, absorbance was determined at 450 nm with a reference set to 630 nm. Background absorbance was determined using wells containing FCM only. Viability was normalized to solvent controls. The experiments were performed with four wells per concentration (technical replicates) and five (SM) or four (H_2_O_2_) times independently (biological replicates).

### Senescence induction after sulfur mustard and hydrogen peroxide exposure

After expansion to approximately 70% confluence in 75 cm^2^ flasks cells were exposed to 3 µM, 13 µM, 24 µM, 40 µM and 65 μM SM or 550 µM H_2_O_2_. SM (8 M) was pre-diluted in pure ethanol (Sigma-Aldrich, St. Louis, USA) and finally diluted in FCM (final ethanol content of 0.33%). The H_2_O_2_ solution (30% [w/w] in H_2_O) was once pre-diluted in ultra-pure water followed by dilution in FCM. Solvent controls were treated with FCM containing 0.33% ethanol. FCM was changed three times a week in the flasks. Cells were passaged as required and at about 70% confluence. For qRT-PCR and Western blot experiments, senescent (65 μM SM) and non-senescent cells (solvent control) were used 21 days after exposure.

### SA‑β‑gal staining for time‑ and concentration dependence of senescence induction

Three independent experiments were performed to evaluate senescence induction in HDF after exposure to solvent control, 3 µM, 13 µM, 24 µM, 40 µM and 65 μM SM or 500 µM H_2_O_2_ in 75 cm^2^ flasks. Development of senescence was monitored every 3–4 days over a period of 31 days using senescence detection kit I (PromoCell, Heidelberg, Germany). Therefore, 10,000 cells were gathered during passaging and grown overnight on coverslips in 24-well plates. In addition, cells were plated onto cover slips in 4-well plates at the same density to exclude false positive staining. According to the manufacturer’s protocol, cells were washed once with PBS, fixed for 10–15 min with the fixative solution at room temperature followed by washing twice with PBS. The senescence staining solution was prepared by mixing 470 μL staining solution, 5 μL staining supplement and 25 μL of 20 mg/mL X-gal in DMSO per well, added to the cells and incubated overnight at 37 °C. Thereafter, cells were washed with PBS, counterstained with nuclear fast red (Vector Laboratories, Inc., Burlingame, USA) for 25 min at room temperature and mounted with VectaMount™ AQ mounting medium (Vector Laboratories, Inc., Burlingame, USA) onto microscopy slides (Thermo Fisher Scientific, Waltham, USA). Three randomly selected images (mean of 20 cells per image) were assessed by TissueFAXS i8 PLUS (TissueGnostics, Vienna, Austria) and the percentage of senescent versus total cells was counted manually.

### *Gene expression *via* qRT-PCR*

21 days after exposure to 65 µM SM and solvent control, 300,000 cells were pelleted by passaging and centrifugation. Total RNA extraction was performed according to RNeasy Kit (Qiagen, Hilden, Germany) and manufacturer’s instructions. In brief, 600 µL buffer RLT was added to the pellet and the cells were lysed under repeated resuspension. The lysate was transferred to a QIAshredder spin column (Qiagen, Hilden, Germany) and centrifuged for 2 min at 21,135×*g*. After mixing the eluate with 600 µL of 70% EtOH, 700 µL of the mixture was added to a spin column followed by centrifugation for 30 s at 13,250×*g*. The column was washed three times, once with 700 µL buffer RW1 (30 s, 13,250×*g*) and twice with 500 µL RPE buffer (2 min, 21,135×*g*). After letting dry the column by centrifugation for 30 s at 21,135×*g*, 30 µL nuclease-free water (Qiagen, Hilden, Germany) were added to column followed by incubation for 1 min at room temperature. RNA was obtained by centrifugation for 1 min at 21,135×*g* in a new biopure 1.5 mL tube (Eppendorf AG, Hamburg, Germany). Using NanoQuant Plate™ with Plate Reader infinite M200 Pro (Tecan Group AG, Männedorf, Switzerland), RNA concentration was determined. cDNA synthesis was conducted with *RT*^2^ First Strand Kit (Qiagen, Hilden, Germany) in accordance with manufacturer’s instructions. Briefly, 500 ng RNA was mixed with 2 µL Buffer GE and RNase-free water to a total volume of 10 µL. The following incubation steps were performed with Mastercycler^®^ nexus GX2 (Eppendorf AG, Hamburg, Germany). After incubation for 5 min at 42 °C, 4 µL 5× buffer BC3, 1 µL control P2, 2 µL RE3 reverse transcriptase mix and 3 µL RNase-free water were added to the mixture and mixed under repeated resuspension. Reverse transcription was performed for 15 min at 42 °C followed by incubation for 5 min at 95 °C. cDNA was diluted with 91 µL nuclease-free water and was stored at − 80 °C for a maximum of 7 days.

For preparation of qRT-PCR plates (Human Cellular Senescence, Human Cell Motility, Human DNA Damage Signalling Pathway, Human Wound Healing [all Qiagen, Hilden, Germany]), 102 µL cDNA was diluted in 1,248 µL RNase-free water. All further pipetting steps were performed with Freedom Evo automated pipetting machine controlled by EVOware™ Standard Software (Tecan Group AG, Männedorf, Switzerland) and repeated incubation with 7% v/v sodium hypochlorite (Carl Roth, Karlsruhe, Germany) in ultra-pure water and washing with ultra-pure water. After transferring 1350 µL of 2 × RT^2^ SYBR^®^ Green ROX qPCR Mastermix (Qiagen, Hilden, Germany) to the diluted cDNA and gentle mixing by inversion, 25 µL of the mixture were added to each well of each qRT-PCR plate. The plate was sealed and placed in the Eppendorf Mastercycler^®^ epgradient S realplex^2^ with Mastercycler ep realplex software (both Eppendorf AG, Hamburg, Germany). qRT-PCR was started by activation of polymerase for 10 min at 95 °C and continued by incubation for 40 cycles of 15 s at 95 °C and 1 min at 60 °C. For analysis, the threshold was set to 200, drift correction was enabled, and *C*_*t*_ values were exported to GeneGlobe (http://www.qiagen.com/geneglobe). For normalization the housekeeping gene RPLP0 was selected, and the following setting were applied: C_t_ cutoff (35), fold regulation cutoff (2) and *p* value cutoff (0.05). The experiment was carried out three times independently.

### Western blot

After exposure to solvent or 65 µM SM and cultivation for 21 days, 300,000 cells, were pelleted and resuspended in 300 µL Tris–EDTA-Triton X-100 extraction buffer (6.25 mM TRIS, 12.5 mM NaCl, 2.5 mM EDTA, 1.5% Triton X-100 [all Sigma-Aldrich, St. Louis, Missouri, USA], one Complete Mini Inhibitor Cocktail and one PhosSTOP [both Roche, Basel, Switzerland] in 10 mL ultra-pure water) followed by incubation on ice for 15 min. Afterwards, cells were sonicated three times for 10 s, 0.3 interval and 30% intensity with ultrasonic homogeniser (Bandelin electronic, Berlin, Germany) and incubated on ice for 1 h. The cell fragments were separated by centrifugation for 10 min at 17,186×*g* at 4 °C. The protein concentration was determined from the supernatant using Qubit™ Protein Assay Kit with Qubit 4 Fluorometer (both Thermo Fisher, Waltham, Massachusetts, USA) in line with the protocol of supplier. The protein lysate was then aliquoted and stored at − 20 °C.

Denaturation was performed by mixing 20 µg protein with 8 µL loading buffer (60% v/v of 4 × Protein Sample Loading Buffer [LI-COR Biosciences, Lincoln, Nebraska, USA] and 3.12% w/v DTT [Sigma-Aldrich, St. Louis, Missouri, USA] in ultra-pure water) and ultra-pure water to a total volume of 25 µL and incubation for 5 min at 95 °C. The mixture was filled in one well of NuPAGE™ 4–12% Bis–Tris gels 1.0 mm × 10 wells with NuPAGE™ MES SDS Running Buffer (both Novex by Thermo Fisher Scientific, Waltham, USA). 3 µL Chameleon™ Duo Pre-stained Protein Ladder (LI-COR Biosciences, Lincoln, Nebraska, USA) was added as molecular weight marker and separation was conducted in a Mini Gel Tank (Invitrogen by Thermo Fisher Scientific, Waltham, USA) for 50 min at 200 V. Protein transfer was carried out with iBlot™ Transfer Stack PVDF (0.2 µm pore size) with iBlot™ 2 Gel Transfer Device (both Thermo Fisher, Waltham, Massachusetts, USA) for 7 min (1 min 20 V, 4 min 23 V, 2 min 25 V) according to the guidelines of the manufacturer. Subsequently, the membranes were blocked for 1 h while gentle shaking in Intercept^®^ (PBS) Blocking Buffer (LI-COR Biosciences, Lincoln, Nebraska, USA). Rabbit Anti-CDKN2A/p16INK4a (ab81278 abcam, Cambridge, UK) 1:2,000 was diluted in antibody diluent (Intercept^®^ (PBS) Blocking Buffer with 0.2% v/v Tween^®^ 20 [Sigma-Aldrich, St. Louis, Missouri, USA]) and added to the membranes for incubation overnight at 4 °C. The next day, antibody diluent was removed by washing for 10 min with washing buffer (PBS with 0.1% v/v Tween^®^ 20), followed by incubation with secondary antibody for 90 min under light exclusion with IRDye^®^ 800CW Goat Anti-Rabbit (LI-COR Biosciences, Lincoln, Nebraska, USA) 1:8000 in antibody diluent. Before using Odyssey CLx with Empiria Studio software for imaging, the membranes were washed again two times for 10 min with washing buffer and rinsed twice with ultra-pure water. Normalization was performed with anti-actin (sc-8432 Santa Cruz, Santa Cruz, USA) primary antibody 1:5000 in antibody diluent and secondary IRDye^®^ 680RD Goat Anti-Mouse (LI-COR Biosciences, Lincoln, USA) 1:8,000 as described above. The samples of two independent biological replicates were analyzed.

### Bio-Plex for secretome analysis

For the generation of samples, HDF, cultured in 75 cm^2^ flasks, were exposed to 24 µM, 65 µM SM, 550 µM H_2_O_2_ or solvent control (day 0). Two days prior to sample collection at days 7, 14, 21 and 28, cells were seeded at 20,000 cells per well in a 24-well plate and grown overnight. The next day, medium was removed and replaced with 600 µL fresh FCM. Precisely 24 h later, the supernatant was collected and centrifuged at 4 °C and 1000×*g* for 15 min. Each sample was divided at 140 µL, filled in four Protein LoBind Tubes (Eppendorf AG, Hamburg, Germany) and stored at − 80 °C.

For sample collection at day 1 after SM, H_2_O_2_ or solvent control exposure, 20,000 cells per well were seeded in a 24-well plate and grown over night. Then, cells were exposed to 24 µM, 65 µM SM, 550 µM H_2_O_2_ or solvent control directly on the 24-well plate (day 0). Exactly after 24 h, the supernatants were collected as described above. For sample analysis, Bio-Plex Pro™ Human Chemokine 40-plex Assay and Bio-Plex Pro™ Human Inflammation 37-plex Assay (both BioRad, Hercules, USA) were used according to manufacturer’s protocol. Washing steps were performed three times (100 μL wash buffer per well) with the wash station HydroFlex (Tecan Group AG, Männedorf, Swiss). IKA^®^ MS3 digital shaker at 850 rpm (IKA, Staufen, Germany) was used for the incubation steps at room temperature, while the plate was covered with aluminum foil. In brief, 50 μL beads were added per well into the Bio-Plex 96-well plate and washed. Standards were diluted according to manufacturer’s protocol and 50 μL of standards or 50 μL of the samples was added per well. After incubation for 1 h, the plate was washed and 25 µL detection antibodies were added per well. The plate was incubated for 30 min. After washing, 50 μL streptavidin–phycoerythrin (SA-PE) was added per well and incubated for 10 min. The plate was washed while beads were resuspended in 125 μL assay buffer. After shaking the plate for 30 s, it was analyzed by the Bio-Plex 200 System and Realplex software (BioRad, Hercules, USA). The experiment was performed in biological duplicates per group and four times independently.

### Statistical analysis

The statistic software RStudio (version 4.0.3 [2020-10-10]) was used for graphic presentation and statistical analysis (RStudio Inc., Boston, USA). Non-linear regression was used for analysis of viability tests and means with 99% confidence interval were presented. For presentation of SA-β-gal results, means and smoothing function with 99% confidence intervals are shown. Apart from that, Tukey boxplots and single data points are displayed. Senescent cells were compared to controls by the unpaired two-sample Wilcoxon test. The *p* values < 0.05 were considered statistically significant. qRT-PCR results are shown as means only for fold regulations > 2.0 or < − 2.0 and *p* < 0.05.

## Results

### ***Cell viability after SM and H***_***2***_***O***_***2***_*** exposure***

**Fig. 1:**
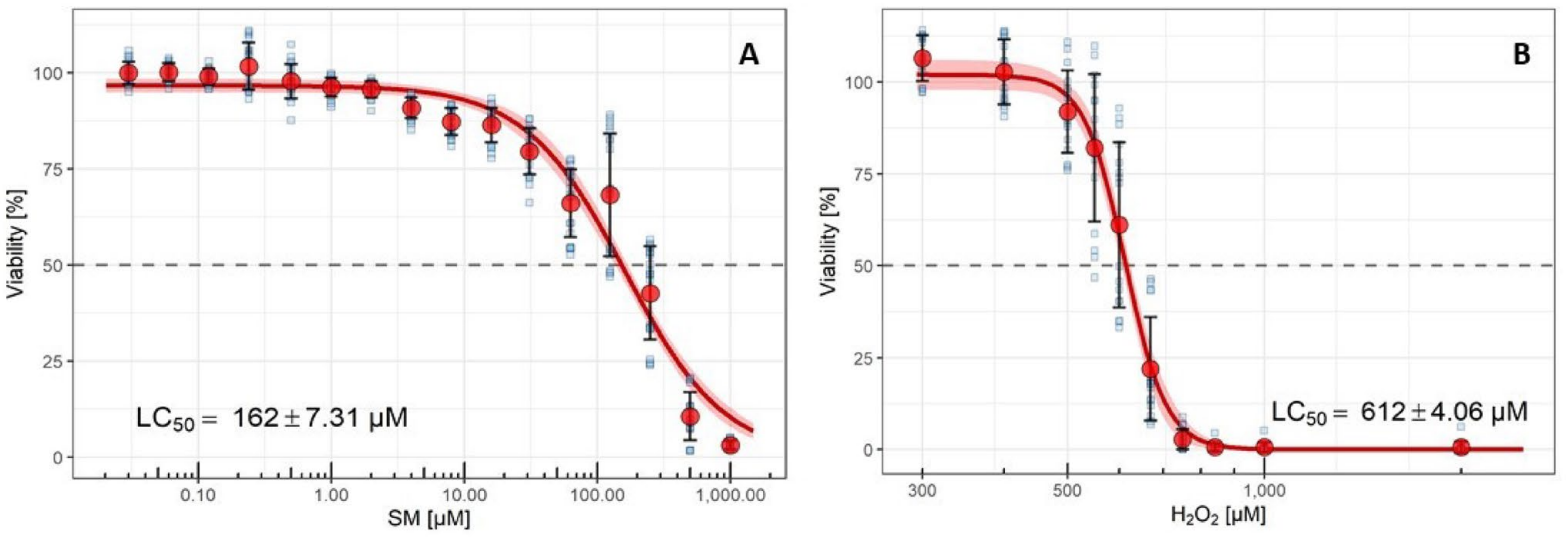
Cell viability after SM and H_2_O_2_ exposure. HDF were exposed to SM (**A**) or H_2_O_2_ (**B**) for 24 h and the XTT assay was used to determine the concentration which led to the death of 50% of SM-exposed HDF (LC_50_). Viability was normalized to corresponding solvent controls. LC_50_ values of 161.7 ± 7.3 μM SM (*n* = 5 biological replicates) and 612.5 ± 4.1 μM H_2_O_2_ (*n* = 4 biological replicates) were determined. Data are represented including mean ± SD (red dots and black error bars), single data points (blue squares), and 99% confidence intervals (red ribbon)

The SM sensitivity of HDF was examined with the XTT assay. HDF were exposed to SM in five independent experiments and cultivated for 24 h. Cytotoxicity was assessed by a broad range of SM concentrations. LC_1_–LC_50_ values were then calculated from the non-linear curve fits. HDF yielded a half-maximal lethal concentration (LC_50_) of 162 ± 7.31 µM (Fig. 1A), indicating a higher tolerance to SM compared to MSC (LC_50_ = 70.7 µM) (Schmidt et al. 2013). Cell survival after H_2_O_2_ exposure was similarly investigated and revealed a LC_50_ of 612.5 µM (Fig. 1B). LC_1_ to LC_50_ values of HDF after SM or H_2_O_2_ exposure are shown in Table 1.

**Table 1:** LC_1_ to LC_50_ values determined by the XTT assay

	SM [μM]	H_2_O_2_ [μM]
LC1	3.1 ± 0.7	439.6 ± 12.4
LC5	12.7 ± 2.0	495.2 ± 9.6
LC10	24.2 ± 3.0	522.6 ± 8.0
LC25	62.6 ± 5.0	565.8 ± 5.7
LC50	161.7 ± 7.3	612.5 ± 4.1

Data are represented as mean ± Std. error

### SA-ß-gal activity as biomarker for senescence after SM exposure

**Fig. 2:**
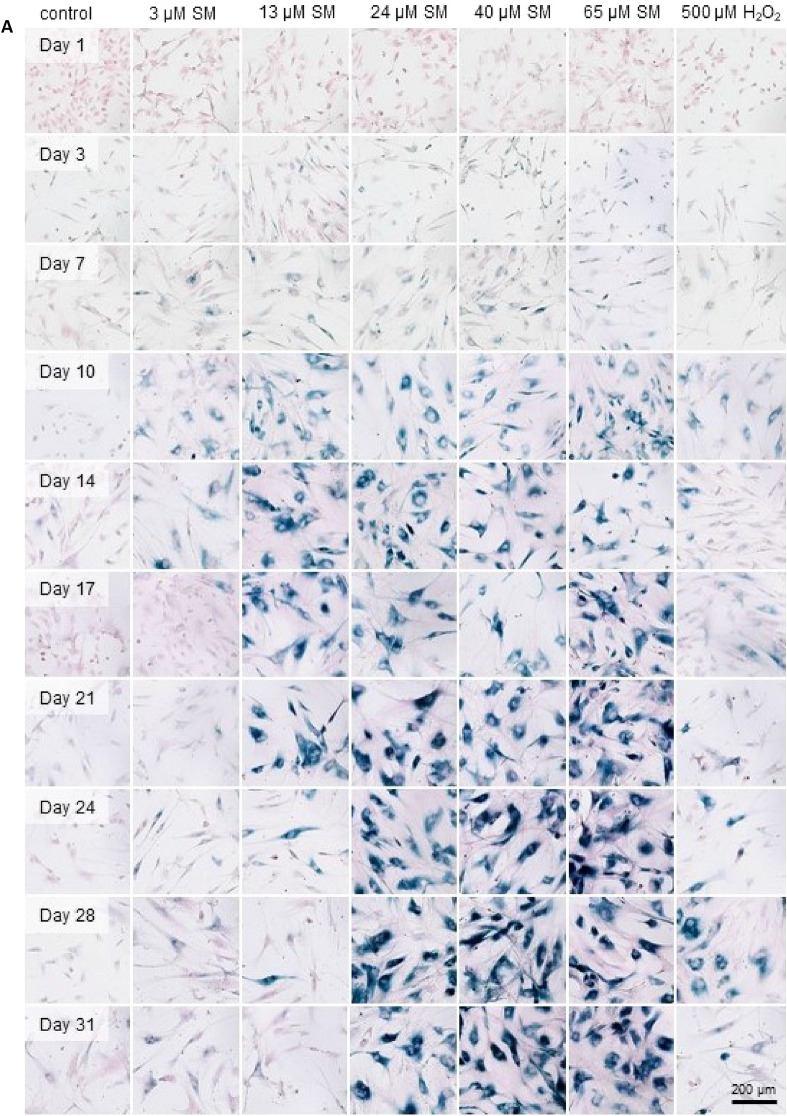

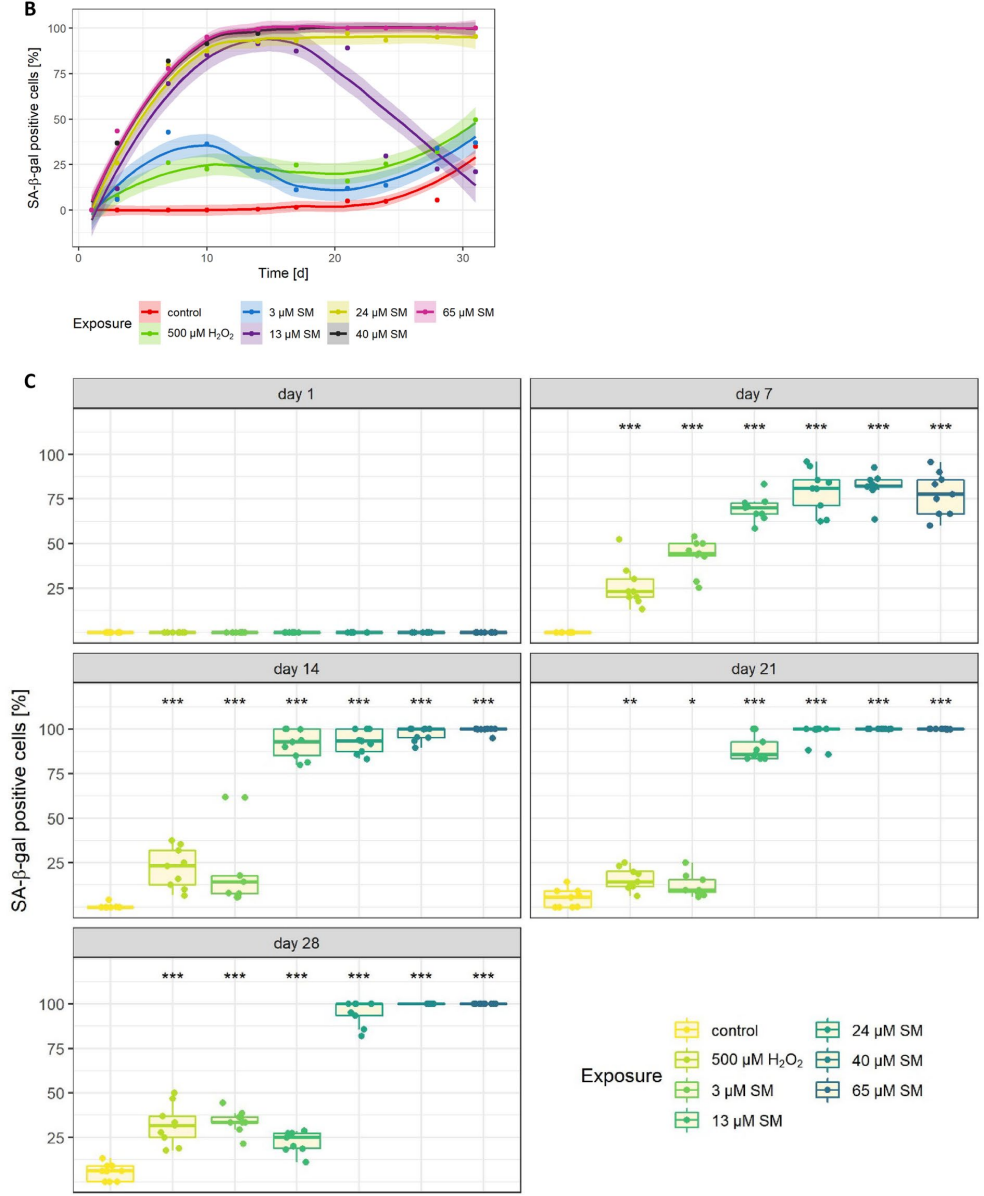
Concentration- and time-dependent SA-β-gal activity. **A** Images of SA-β-gal staining (blue) after single-dose exposure to solvent control (control), SM or H_2_O_2_ at day 0. Cells were counterstained with nuclear fast red (red). Stable SA-β-gal activity was induced by 24 μM SM, 40 μM SM or 65 μM SM. SA-β-gal induction after exposure to 3 μM SM or 13 μM SM was not persistent. 500 μM H_2_O_2_, used as positive control for SA-β-gal activity, was insufficient for stable induction. SA-β-gal-positive cells showed an increased cell size. Scale bar, 200 μm. **B** Percentage of SA-β-gal-positive cells counted over 31 days. Three randomly selected picture sections per group (see **A**) from three independent experiments were analyzed. Data are represented as means with 99% confidence intervals (colored ribbons). **C** Senescence induction depicted by SA-β-gal activity in HDF by SM or H_2_O_2_ exposure. Percentage of SA-β-gal-positive HDF 1–28 days post-exposure to solvent control, SM and H_2_O_2_ (*n* = three randomly selected picture sections per group from three independent experiments). The significance of each group compared to corresponding solvent controls is illustrated. Data are represented as boxplots; *p < 0.05, **p < 0.01, ***p < 0.001

Sub-lethal concentrations of SM were used to induce senescence in HDF. The lethal concentrations for 1%, 5%, 10%, 17% and 25% were set at 3 µM, 13 µM, 24 µM, 40 µM and 65 µM SM, respectively, based on the results of the XTT assay. H_2_O_2_ has been reported to induce a senescent-like state in fibroblast cells (Chen and Ames 1994) and was thus used as positive senescence induction control. Hence, 500 µM H_2_O_2_ (LC_6_) was used for all further experiments. To determine the percentage of senescent cells over time, SA-β-gal staining was performed up to 31 days after SM exposure (day 0) at 3–4-day intervals. Figure 2A–C shows an increase of staining intensity and percentage of senescent cells with rising SM concentrations over time. Exposure to 24 µM, 40 µM or 65 µM SM resulted in a stable senescent state from day 14 to day 31. In contrast, exposure to 13 µM SM revealed only a transient senescent state within day 10 to 21. Cells seemed to re-enter the cell cycle as a decrease of SA-β-gal staining intensity occurred after day 21. In general, induction of senescence after H_2_O_2_ exposure was not as strong and stable as after SM exposure. Cells exposed to solvent control did not show a relevant increase in senescence over time.

### Upregulated genes and markers associated with senescence

**Fig. 3:**
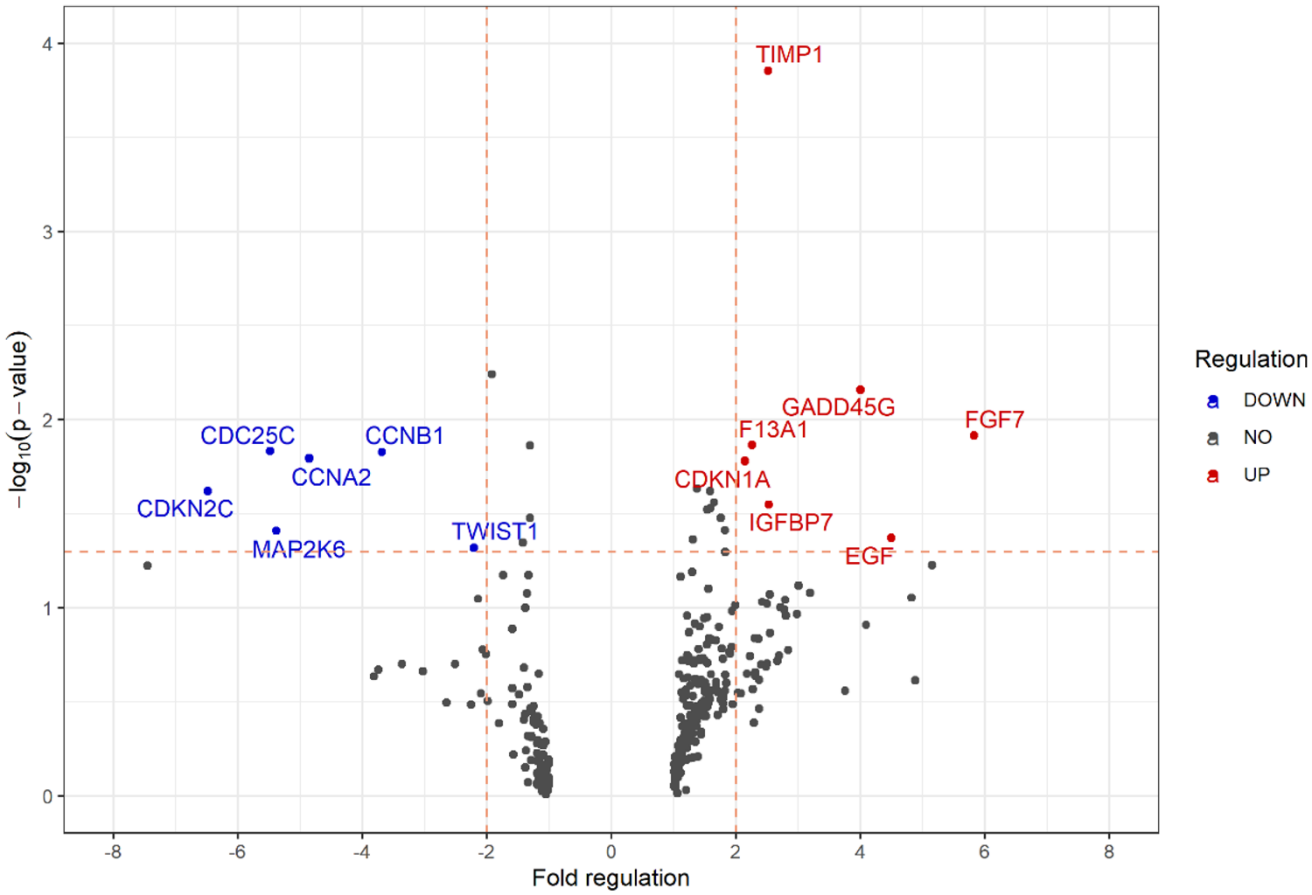
Changes on the gene level. After 21 days of 65 μM SM exposure, significant regulation of genes (fold regulation > 2.0 or < − 2.0 in combination with *p* < 0.05) associated with senescence, DNA damage and repair was determined by qRT-PCR in comparison to solvent control (control). Data are shown as means (*n* = three independent experiments). The horizontal dashed line is applicable for a *p* value of 0.05

**Fig. 4:**
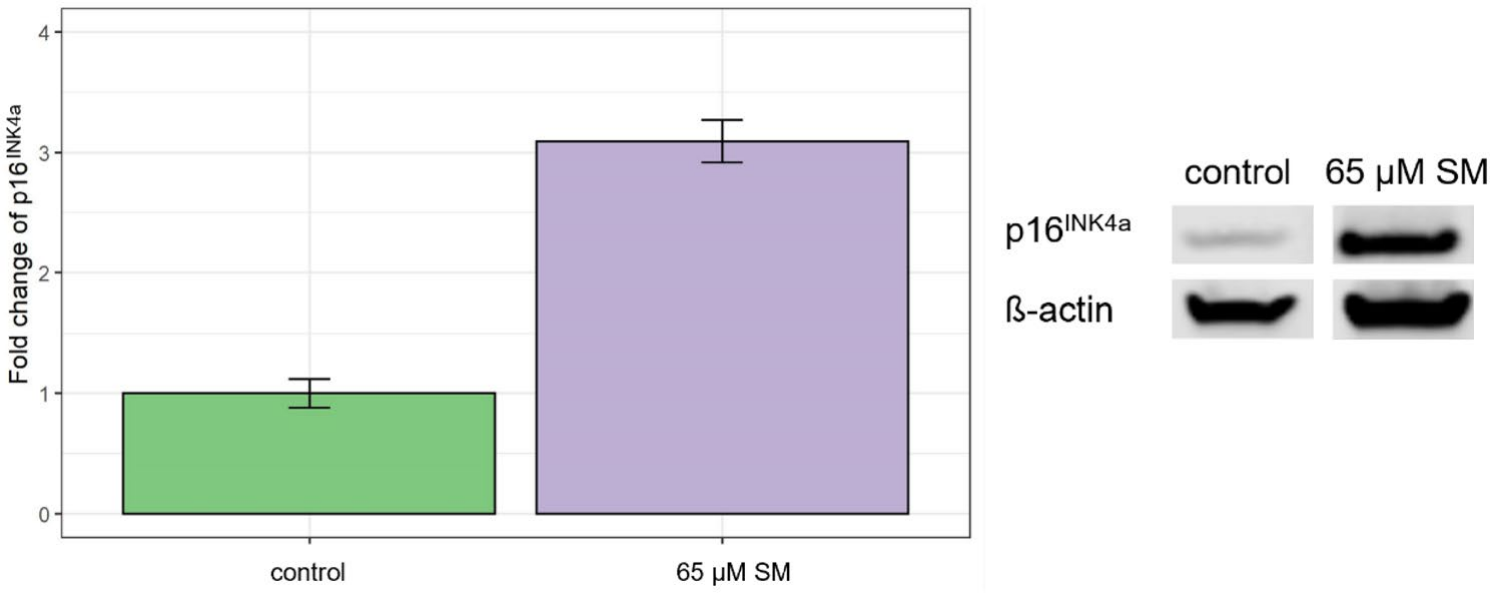
Changes on the protein level upregulation of p16INK4a in comparison to solvent control (control) was observed using Western blot. Fold changes ± scattering and representative bands are shown (*n* = two independent experiments)

Significant differences in expression of genes (fold regulation > 2 or < − 2 in combination with *p* < 0.05) and protein were observed in cells exposed to 65 µM SM compared to cells exposed to solvent control using qRT-PCR and Western blot. As shown in Fig. 3, some genes such as ‘fibroblast growth factor 7 (FGF7)’, ‘epidermal growth factor (EGF)’ and ‘growth arrest and DNA damage inducible gamma (GADD45G)’ were more upregulated (fold regulation ≥ 4.0) as other genes such as ‘insulin like growth factor binding protein 7 (IGFBP7)’, ‘TIMP metallopeptidase inhibitor 1 (TIMP1)’, ‘coagulation factor XIII a chain (F13A1)’ and ‘cyclin-dependent kinase inhibitor 1A (CDKN1A, p21 ^WAF/Cip1^)’. In comparison, ‘cyclin A2 (CCNA2)’, ‘mitogen-activated protein kinase kinase 6 (MAP2K6)’, ‘cell division cycle 25C (CDC25C)’ and ‘cyclin dependent kinase inhibitor 2C (CDKN2C, p18)’ were detected to be strongly downregulated (fold regulation ≤ − 4.0). In addition, ‘Cyclin B1’ (CCNB1)’ and ‘twist family bHLH transcription factor 1 (TWIST1)’ were also observed to be significantly downregulated. By means of Western blot analysis, ‘cyclin-dependent kinase inhibitor 2A (CDKN2A, p16^INK4a^)’ was found increased by threefold changes when compared to solvent control (Fig. 4).

### Secretion of pro-inflammatory factors due to senescence

**Fig. 5:**
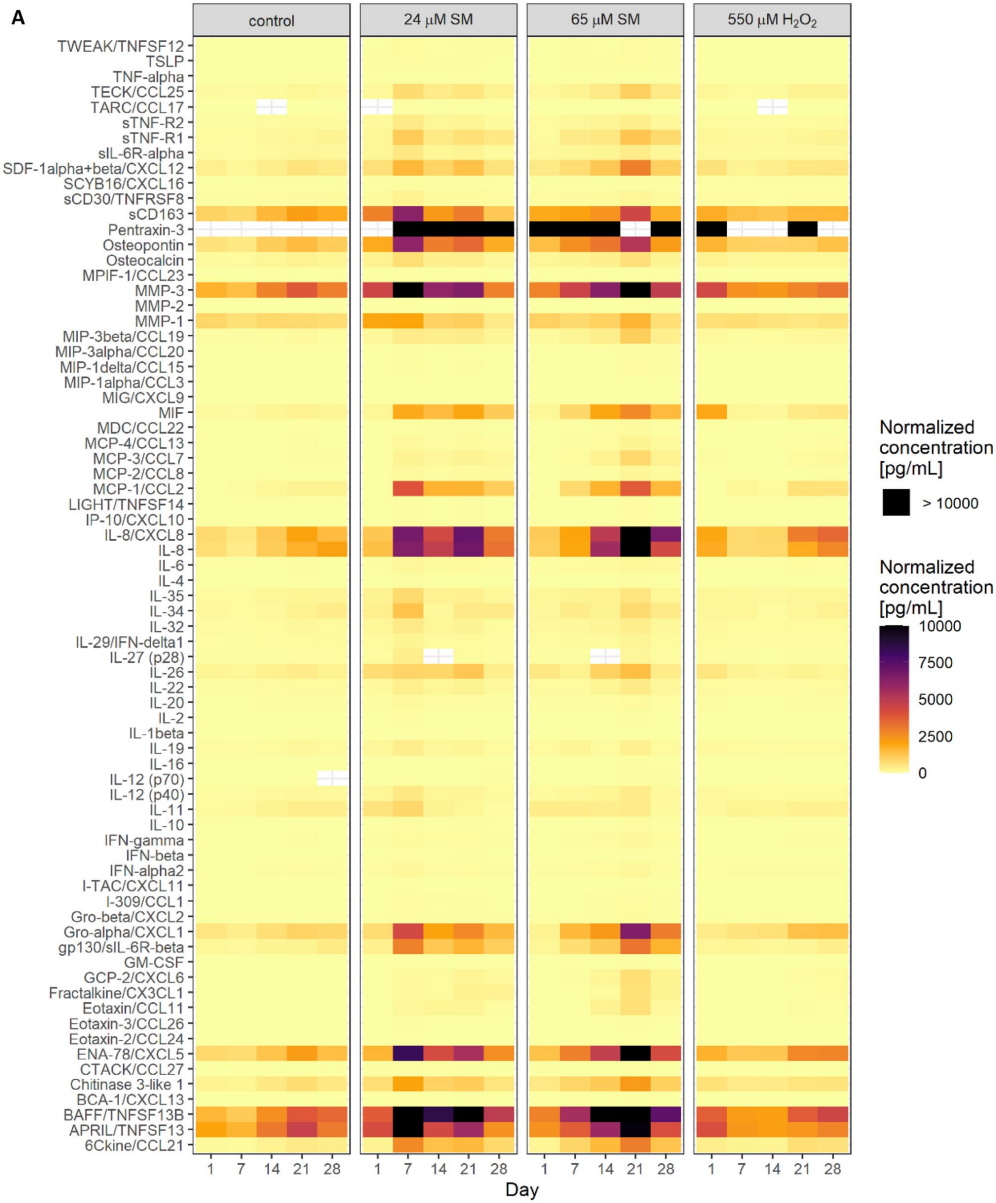

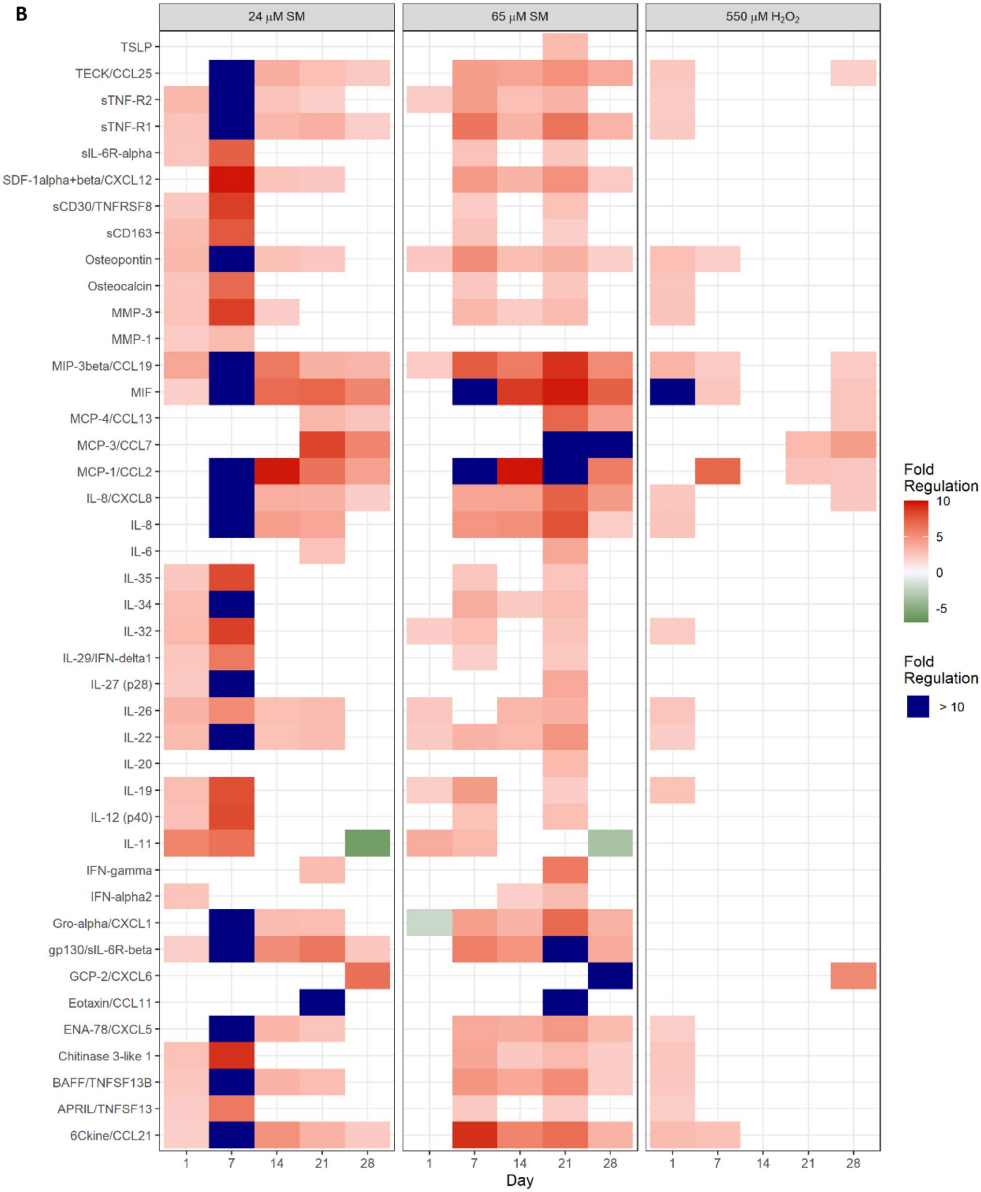
Secreted factors in cell culture supernatants. **A** Cell culture supernatants were collected 24 h after cell seeding at days 1, 7, 14, 21 and 28 after solvent (control), SM or H_2_O_2_ exposure. The Bio-Plex assay was used to determine the concentration of 73 chemokines, cytokines and growth factors. Concentrations were normalized for the respective cell numbers. Data are represented as means of normalized concentrations. Missing color indicates levels below the detection limit (CCL 17, IL-27, IL-12) or above the detection limit (Pentraxin-3), *n* = biological duplicates per group from three independent experiments. **B** Fold regulation of up- or downregulated factors (> 2.0 or < − 2.0 in combination with normalized concentrations above 20 pg/mL) in SM- and H_2_O_2_-exposed HDF in comparison to solvent control are shown

To point out differences in the secretome of senescent HDFs, 73 different growth factors, chemokines and cytokines were analyzed by Bio-Plex assays. As the SASP is a time-dependent, dynamic process, levels of secreted factors were determined at days 1, 7, 14, 21 and 28 after exposure to solvent control, 24 µM and 65 µM SM or 550 µM H_2_O_2_ (Fig. 5A). Fold regulation was calculated for all factors compared to the corresponding solvent control. Some factors were generally secreted at small levels (e.g., GM–CSF or I–309/CCL1; ≤ 20 pg/mL) or fold regulation (*f*_r_) was low (e.g., IL-1β or IL-4; − 2 ≤ *f*_r_ ≤ 2). In Fig. 5B, factors were selected which showed a fold regulation > 2 or < −2 and were secreted at levels > 20 pg/mL. Many factors such as e.g., IL-8, CCL2 or Gro-α were especially highly expressed from day 7 and are associated as factors of a “full” SASP (Coppé et al. 2010). In addition, many other chemokines, and cytokines (e.g., CCL19, MIF, CXCL12, gp130, sTNF-R1, sTNF-R2, Osteopontin, MMP-3 or CCL21) were found upregulated at almost all timepoints after SM exposure and can thus be designated as constant factors. Some factors, e.g., IL–11, IL-19 or IL-35 were secreted early during the first week after SM exposure but were subsequently found around control levels or even downregulated. MCP–4/CCL13, MCP–3/CCL7 and CXCL6 can be termed as late factors and were upregulated 21–28 days after SM exposure. Levels of Pentraxin-3 were > 10.000 pg/mL or above the detection limit in all samples. Concerning H_2_O_2_ exposure, secretion of factors was not stable, and in general, upregulation occurred with fewer factors and to a lesser degree.

## Discussion

In this study, the effects of single-dose SM exposure on HDF were investigated which provides new insight into the pathomechanism of SM-associated wound healing disorder. Previously published research demonstrated that senescent fibroblasts play an important role in chronic wounds and that single-dose SM exposure induces senescence in MSC which may also contribute to chronic wound healing disorders (desJardins-Park et al. 2018; Rothmiller et al. 2021). The results of this study hereby confirm for the first time that senescence can also be induced in HDF by SM exposure in a time- and concentration-dependent manner which was detected using different markers of senescence. To omit cytotoxic effects by SM exposure, only SM concentrations such as LC_25_ or lower were applied for all experiments (Fig. 1). As a result, the appearance of HDF changed into a flattened and enlarged morphology which is a specific phenotype of senescent cells (Fig. 2A) (Ho and Dreesen 2021). Furthermore, SA-ß-gal activity is considered as one of the biomarkers for senescence as senescent cells highly express the lysosomal enzyme ß-galactosidase (Faragher 2021). HDF exposed to 24 µM SM, 40 µM SM or 65 µM SM showed a stable senescence from day 14 as a constant intensity of SA-ß-gal staining was detected (Fig. 2B).

Three other factors most commonly used to validate senescence in combination with SA-ß-gal are p16^INK4A^, p21^WAF/Cip1^ and CCNA2 (Gorgoulis et al. 2019). The cell cycle kinase inhibitor proteins p16^INK4A^ and p21^WAF/Cip1^ are responsible for the induction and maintenance of senescence by inhibition of cell cycle progression through indirect inhibition of E2F transactivation (Wlaschek et al. 2021; Gorgoulis et al. 2019; Rayess et al. 2012). Previous investigations found p16^INK4A^ and p21^WAF/Cip1^ being increased expressed in dermal fibroblasts undergoing replicative senescence and upon ultraviolet radiance exposure (Ho and Dreesen 2021). Furthermore, upregulation of p16^INK4A^ and p21^WAF/Cip1^ was also observed in MSC after SM exposure (Rothmiller et al. 2021). These observations are related to our results as SM-induced senescent fibroblasts showed increased expression of p21^WAF/Cip1^ (CDKN1A) on the mRNA level (Fig. 3) and of p16^INK4A^ on the protein level (Fig. 4). A recent study revealed that CCNA2 mRNA is an important regulator of senescence by being part of the newly identified p53/miRNAs/CCNA2 pathway. Silencing of CCNA2, as also observed here (Fig. 3), induces senescence, as it can no longer be affected by miR-29 and miR-124 which act as antagonists of p21^WAF/Cip1^ (Kumari and Jat 2021).

In addition to cell cycle arrest, senescent fibroblasts are also characterized by a uniquely altered and temporally dynamic secretome, their senescence-associated secretory phenotype (SASP), which is triggered by e.g., DNA damage response (DDR) which in turn is described being activated by SM exposure (Wlaschek et al. 2021; Kumari and Jat 2021; Schmidt et al. 2018). Consistent with these findings, we identified cytokines and chemokines typically associated with the SASP (Coppé et al. 2010). CCL25, CXCL12, CCL2, IL-8, Gro-α/CXCL1, CXCL5 and gp130 can be termed as factors of a “mature” SASP as they were secreted at a high level from day 7 onwards. Furthermore, CCL19, MIF, sTNF-R1, sTNF-R2, Osteopontin (OPN), MMP-3, IL-22, BAFF, TNFSF13B and CCL21 can be considered as constant factors as they were highly expressed at multiple time points (Fig. 5B).

A key modulator of SASP and inducer of senescence, IGFBP7 was found significantly upregulated at the mRNA level (Fig. 3) and is associated with growth arrest (Severino et al. 2013). Surprisingly, the only early expressed and later downregulated factor was IL-11 (Fig. 5B) which has already been identified as a pro-fibrotic factor and promoter of fibroblast senescence (Adami et al. 2021; Kumari and Jat 2021; Ng et al. 2020). Determination of p16^INK4A^ expression level at the time of observed IL-11 downregulation is part of further studies as both senescence factors seem to be linked via TGF-β1/IL-11/MEK/ERK (TIME) signaling pathway and permanent upregulation of p16^INK4A^ is essential for the maintenance of senescence (Chen et al. 2020; Kumari and Jat 2021).

Another major factor, besides IL-8, characterizing the inflammatory SASP is CCL2 which was found highly expressed after SM exposure and is thought to play a key role in chronic inflammatory diseases (Kumari and Jat 2021; Barrientos et al. 2008). CCL2 might also be relevant in line with CXCL12 and late expressed factors such as CCL7, being described as enhancing cancer cell migration, CXCL6, being associated with promoting migration and proliferation of cancer cells, and CCL13 in terms of neovascularization and cancer (Mukaida et al. 2014; Hao et al. 2020; Jung et al. 2010; Coppé et al. 2010; Korbecki et al. 2020; Liu et al. 2019). In this context, upregulated growth factors such as FGF7 and EGF, also described being expressed in SASP of senescent fibroblasts, in combination with CCL2, CCL7, CCL13, CXCL6 and CXCL12 might play an important role in cancer progression which is relevant in that neoplasms can occur as late effects after SM exposure (Poursaleh et al. 2012; Coppé et al. 2010; Steinritz 2016). In addition, IL-35, normally not regulated in the SASP, was found upregulated in SM-exposed HDF which might also promote proliferation and angiogenesis in cancer (Kourko et al. 2019; Byun et al. 2015). Thus, these factors might be potential targets for anti-cancer therapy after SM exposure.

Many of the here listed SASP factors (e.g., IL-8, OPN, gp130, sTNF-R1 or MIF) and senescence-related cell cycle regulators being upregulated (CDKN1A, GADD45G, TIMP1) and downregulated (CCNB1, CCNA2, CDKN2C) coincide with results of regulated genes and proteins in SM-exposed and chronic senescent MSC (Rothmiller et al. 2021). Interestingly, SM-induced senescence of both cell types result in multiple similarly affected SASP factors and senescence-associated genes suggesting some kind of equivalent cellular answer to these toxic effects by SM. Especially, these could provide new drug targets to counteract chronic wound healing in a broader context.

Newly identified factors regulated after SM exposure are sTNF-R2, TWIST1, CDC25C, MAP2K6, F13A1 and CCL19. It is described that downregulation of CDC25C induces cell cycle arrest in response to DNA damage and that downregulation of MAP2K6 (MKK6) is associated with anti-apoptotic effects (Rahman et al. 2020; Liu et al. 2020). Thus, both factors seem to promote senescence in HDF after SM exposure. Furthermore, an increased proton leak was found related to reduced TWIST1 expression in MSC suggesting mitochondrial dysfunction which is a potential senescence inducer (Voskamp et al. 2021). An altered mitochondrial phenotype in SM-exposed skin samples as well as an increase in mitochondrial mass in senescent fibroblasts was observed which also indicates a disorder of mitochondrial function (Voskamp et al. 2021; Kehe et al. 2009). Investigations on mitochondrial alterations in context of SM-induced senescence in HDF are, therefore, part of ongoing studies. The specific role of upregulated sTNF-R2, CCL19 and F13A1 remains elusive. However, sTNF-R2 was detected in the extracellular milieu of senescent fibroblasts and F13A1 is considered to be involved in inflammatory as well as in wound healing processes and seems to enhance dermal fibroblast proliferation as well as migration of dendritic cells in synergy with CCL19 (Paragh and Törőcsik 2017; Kumar et al. 2014).

The expression of SASP and senescence regulating factors differs dependent on, i.e., the cell type and the affecting senescence inducer (Kumari and Jat 2021). Therefore, IL-6 or IL-1B/GM-CSF being part of the characteristic inflammatory SASP and not being upregulated in this study might be due to SM as a novel senescence inducer in fibroblasts (Kumari and Jat 2021). Furthermore, our results indicate that H_2_O_2_ is not adequate as a positive control for the detection of senescence in HDF as neither SA-ß-gal staining nor expression of SASP factors was stable, although it is described to induce a senescence-like growth arrest in human diploid fibroblast (Chen and Ames 1994).

To sum up, our results confirm for the first time that single-dose SM exposure induces senescence in HDF, compromising their proliferation and leading to the secretion of various cytokines and chemokines. In consequence, senescence might be triggered in neighboring cells which in turn increases the risk of impairment in fulfilling their role in the wound healing process. Altogether, this might contribute to chronic wound healing disorder and carcinogenesis as late effect of SM exposure.

Further investigations are needed to identify the type and pathomechanism of SM-induced senescence in HDF. Ongoing studies concerning the effects of senolytic substances on senescent HDF such as navitoclax (ABT-263), which is described to eliminate senescent lung fibroblasts and SM-induced chronic senescent MSC, might help to find new therapeutic approaches for the SM-induced wound healing disorder (Rothmiller et al. 2021; Wlaschek et al. 2021).
